# Manifestation of Hyperandrogenism in the Continuous Light Exposure-Induced PCOS Rat Model

**DOI:** 10.1155/2015/943694

**Published:** 2015-05-03

**Authors:** Xuezhi Kang, Lina Jia, Xueyong Shen

**Affiliations:** ^1^College of Acupuncture and Moxibustion, Shanghai University of Traditional Chinese Medicine, Shanghai 201203, China; ^2^Shanghai Research Center for Acupuncture and Meridians, Shanghai 201203, China; ^3^Department of Traditional Chinese Medicine, Huadong Hospital, Fudan University, Shanghai 200040, China

## Abstract

Polycystic ovary syndrome (PCOS) is a complex endocrine and metabolic disorder, and its pathogenesis has yet to be completely clarified. A fully convincing animal model has not been established for PCOS. In earlier studies, researchers have shown that the exposure of rats to continuous light can induce PCOS; nevertheless, hyperandrogenism, a key characteristic observed in human PCOS, has not been reported previously. In the present study, we found that (1) body weights decreased in female rats in a continuous light environment with both ovarian and uterine augmentation; (2) the estrous cycle in rats under continuous light environment was disordered, and polycystic ovary-like changes occurred, accompanied with fur loss and lethargy; and (3) serum testosterone levels in rats in a continuous light environment significantly increased. Our data suggest that continuous light can lead to the occurrence of PCOS in female rats without the need for drugs; this is a reasonable PCOS animal model that is more consistent with the natural disease state in humans; and poor sleep habits or negligence of sleep hygiene may be an important lifestyle factor in pathogenesis of PCOS.

## 1. Introduction 

Polycystic ovary syndrome (PCOS) is a complex endocrine and metabolic disorder associated with ovulatory dysfunction, abdominal obesity, and infertility, and it affects 5%–10% of women of reproducing age [1]. In 1935, Stein and Leventhal published a case series of seven women with amenorrhea and bilateral polycystic ovaries. At the National Institutes of Health (NIH) consensus conference held in 1990, PCOS was defined as chronic anovulation with clinical and/or biochemical hyperandrogenism, excessive androgen secretion or activity; clinical manifestations of acne and hirsutism are the primary characteristics of PCOS [2]. The pathogenesis of PCOS is uncertain, although lifestyle may be an important factor. In recent years, the understanding of the pathogenesis of this disease has significantly advanced; however, a fully convincing animal model for PCOS has not been established [3]. Depending on which PCOS-related disorder is investigated, the most suitable animal model should be utilized.

Voluntary sleep restriction has become widespread in modern society [4]. In humans, chronic partial sleep loss and behavioral and sleep disorders have been linked with obesity and metabolic syndrome [5]. Many women with PCOS have psychological problems and sleep disorders that significantly reduce sleep quality [6]. The risk for obstructive sleep apnea (OSA) is higher in obese women with PCOS [7]. In female mammals, ovulation is induced by luteinizing hormone (LH) secretion surge from the pituitary gland, an event which is itself stimulated by gonadotropin releasing hormone (GnRH) secretion from neurons in the hypothalamus [8]. In rodents, the LH surges that trigger ovulation are controlled by cyclic light-dark photoperiods [9]. A disturbance of these light-dark photoperiods within a 24-hour period can disrupt normal cycling in rats and inhibit ovulation [10]; exposure of female rats to a constant light environment was developed as an alternative approach for inducing PCOS [11, 12].

According to the literature, PCOS can be induced in rats in fewer than 75 days of continuous exposure to 600-lux light [12]. Research on the constant light modeling method was published in the 1970s. Due to the limitations of the experimental conditions and the awareness of PCOS [13], hyperandrogenism, a key characteristic observed in human PCOS, was not reported in this model [14]. The purpose of this study was to determine the level of testosterone in the rat model and reevaluate the constant light exposure-induced PCOS rat model with a contemporary perspective to unequivocally provide a PCOS animal model for future research that is more consistent with the natural disease state.

## 2. Materials and Methods

### 2.1. Animals

All animal procedures were performed in accordance with the guidelines of the Animal Care and Use Committee of Huadong Hospital and Shanghai Research Center for Acupuncture and Meridians. Six-week-old female Sprague Dawley (SD) rats were purchased from the Shanghai Experimental Animal Center of Chinese Academy of Sciences and were housed at 22–25°C in a custom-designed experimental box. All rats were allowed to eat and drink freely.

### 2.2. Equipment and Reagents

The custom-designed light experiment box had a length, width, and height of 120 cm, 45 cm, and 180 cm, it was vertically divided into four equal and independent chambers (length, width, and height of 120 cm, 45 cm, and 45 cm for each chamber) with independent ventilation. A fluorescent lamp (color temperature: 6500 K, illumination: 600 lux) was installed in every chamber and had lights controlled by a microcomputer switch that allowed free adjustment of the illumination time. This study also utilized a microscope (Nikon, ECLIPSE Ti-S, Japan) and microplate reader (Thermo, Multiskan FC, China); all other chemicals and reagents were purchased from the China National Pharmaceutical Group Corporation.

### 2.3. Continuous Light/Dark

The female rats were randomly divided into control and experimental groups and placed in the light experiment box. The control group was under a circadian rhythm of 12:12 h light-dark cycle (L/D, lights on at 8 a.m. Beijing standard time). The rats in the experimental group were exposed to a continuous light environment (L/L, lights on 24 hours every day) for 16 weeks.

### 2.4. Vaginal Smears

Daily vaginal smears were performed on all rats, and their estrous cycles were observed. A sterile cotton swab was soaked in 0.9% saline before it was smeared around the first 1/3 of the vaginal wall. The cotton swab was removed and was smeared in the same direction on a glass slide. The cells were evaluated under light microscopy, and the samples were classified as 1 of the 4 stages of the estrous cycle. Diestrus vaginal smears were identified by the presence of high numbers of leukocytes, proestrus vaginal smears were identified by the presence of small-nucleated epithelial cells, estrus smears were identified by large numbers of cornfield epithelial cells, and metestrus smears were identified by the presence of leukocytes.

### 2.5. ELISA Analysis

At the end of 16 weeks, the rats were anesthetized, and blood samples were obtained from the inferior vena cava and placed into Vacutainer tubes with coagulant. Serum was separated and stored at −80°C for subsequent testosterone determination by enzyme-linked immunosorbent assay (ELISA) (Testosterone EIA Kit, Cayman Chemical, item number 582701, made in USA). ELISA was performed according to the instruction manual of the EIA kit.

### 2.6. Histology

Ovary tissue from all rats was harvested after euthanasia. After weighing with a precision balance (Sartorius, BT 125D, Germany), the ovaries were fixed with 4% formaldehyde buffer, embedded in paraffin, sectioned into 4 *μ*m slides, and stained with hematoxylin-eosin (H&E). The sections were observed, and photographs were taken using a Nikon microscope (CLIPSE Ti-S, Japan).

### 2.7. Statistical Analysis

Data are presented as the means ± SE. Statistical significance was determined using either Student's *t*-test or Fisher's exact test. Statistical significance was defined as *P* < 0.05, and two-tailed tests were used. All data analysis and graphing were carried out using Origin 8.0 software (Origin Lab, USA) or SPSS 19.0 for Windows.

## 3. Results

### 3.1. Continuous Light-Induced Changes in Body, Ovarian, and Uterus Weights

**Figure 1:**
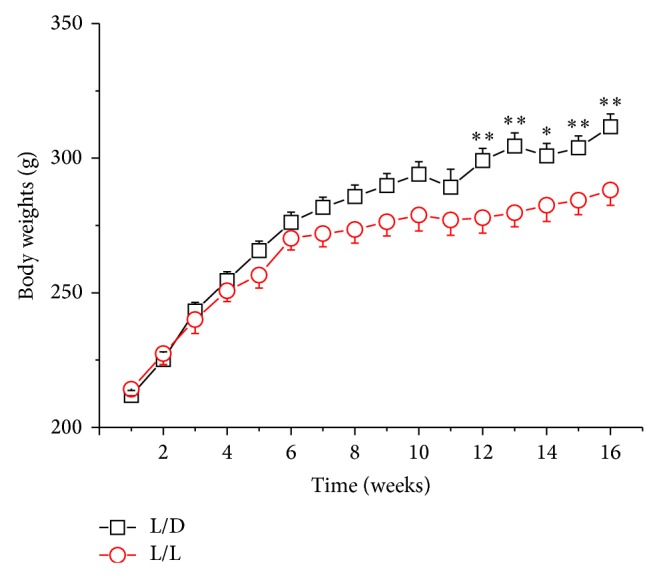
The changes of rats' body weights; *n* = 24, with 12 rats in each group. ^∗^ *P* < 0.05, ^∗∗^ *P* < 0.01 L/L group versus control (L/D).

Changes in body, ovarian, and uterine weight were monitored in all animals. Compared with the control group, body weight growth in L/L rats became slow after 5 weeks (body weights of L/L group versus control at the end of 5 weeks: 256.56 ± 4.85 g versus 265.67 ± 3.61 g, *P* > 0.05). After 12 weeks, the difference of body weights in two groups was statistically significant (body weights of L/L group versus control at the end of 12 weeks: 277.91 ± 5.76 g versus 299.08 ± 4.60 g, *P* < 0.01) and persisted for the remainder of the experiment (Figure 1).

**Figure 2:**
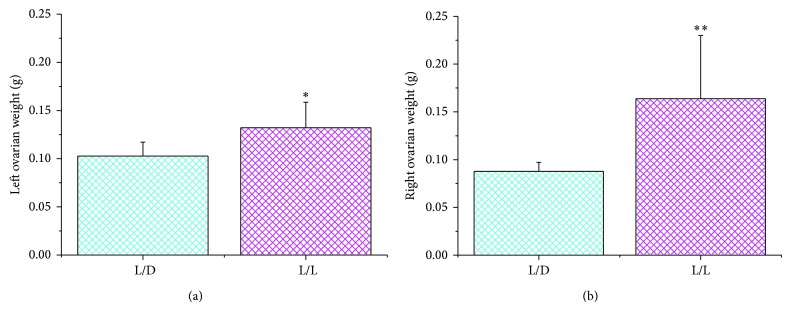
The left ovarian weights (a) and right ovarian weights (b) in the two groups. There was a statistically significant difference between them (^∗^ *P* < 0.05, ^∗∗^ *P* < 0.01 L/L group versus control).

**Figure 3:**
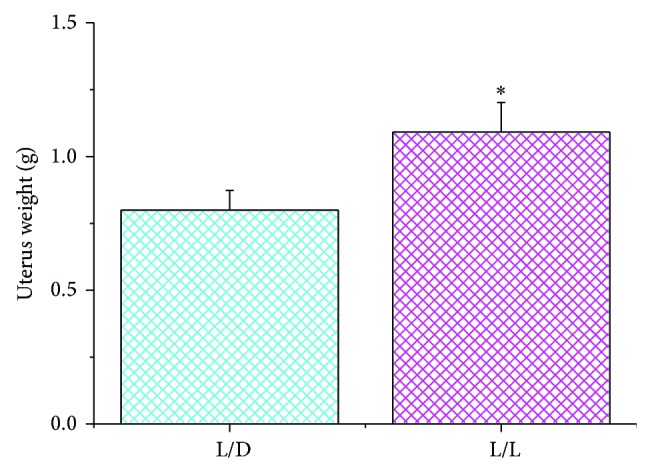
The average uterine weight in the two groups (^∗^ *P* < 0.05 L/L group versus control).

We compare the weight of the bilateral ovaries: either the left ovary or the right ovary in the L/L group was heavier than that in the L/D group (left ovary, *P* < 0.05; right ovary, *P* < 0.01) (Figure 2). Similar to the weight of the ovaries, hypertrophied uteri were present in the L/L rats. Uterine weights in the L/L rats were higher than those in the L/D group (Figure 3) (*P* < 0.05).

### 3.2. Changes in the Estrous Cycle

**Figure 4:**
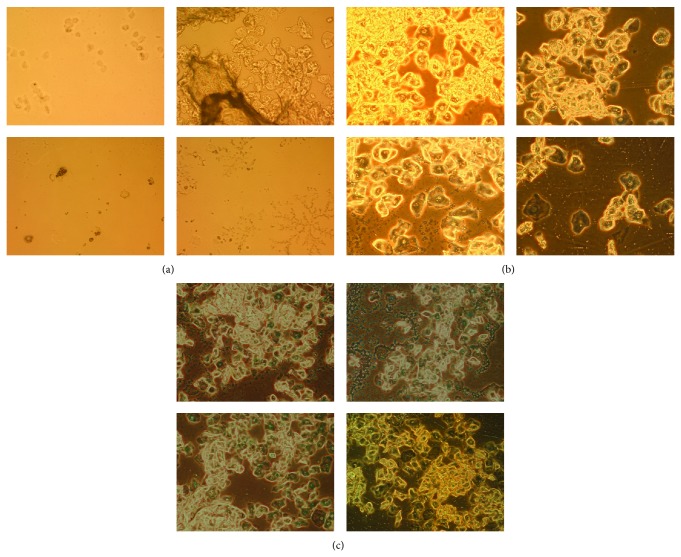
Typical vaginal smear for four consecutive days in different experimental periods and different groups. (a) Normal rat's vaginal smear; (b) L/L group in the 4 weeks; (c) L/L group in the 16 weeks. Magnification is 100x.

Using daily vaginal smears, estrous cyclicity was analyzed at the beginning, after 4 weeks and after 16 weeks of the experimental process.  Figure 4 shows the following results: (1) before continuous light, vaginal smears on four consecutive days show a disciplinary transmutation with a sequence of diestrus, proestrus, estrus, and metestrus (Figure 4(a)) and (2) at the end of 4 weeks, 8 of the 12 rats (66.7%) (Table 1) in the continuous light group displayed an indiscriminate estrous cycle. We observed that, in the vaginal smears, the cyclicity stopped at a proestrous state on all four days (Figure 4(b)). Using Fisher's exact test, the difference was significant (*P* = 1.35*E* − 3). (3) At the end of 16 weeks, all the L/L rats (12 of 12, 100%) showed an indiscriminate estrous cycle (Figure 4(c)), similar to that after 4 weeks; and 1 of 12 rats in control group also showed an indiscriminate estrous cycle (Table 2, L/D versus L/L *P* = 9.61*E* − 6).

### 3.3. Changes in Fur and Mental Status

**Figure 5:**
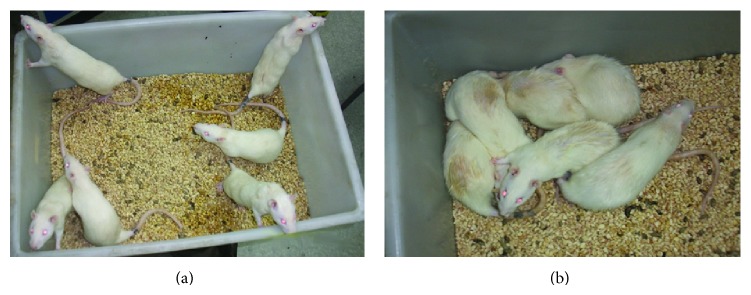
Apparent states among rats in the continuous light group at the end of 16 weeks. (a) In the control group the rats were in good spirits and were agile, and their fur was clean and smooth. (b) In the continuous light group the rats showed a poor mental state, were lethargic, and had decreased activity, and their fur, especially on the lower back, was dirty and showed obvious loss.

Similar to human PCOS, L/L rats demonstrated patches of diffuse fur loss, and their furs were unburnished grey compared to the shiny fur of the control group. Loss of fur from the dorsal and neck portions of the rats was clearly visible. The difference in fur loss in each group was evaluated by visual observation and was recorded by photographs (Figures 5(a) and 5(b)). All rats in the L/L group showed different degrees of depilation; some rats had serious loss on the back and neck, and some had loss on the hip. At the same time, in the continuous light group, all rats showed a poor mental state, exhibiting lethargy and decreased activity (Figure 5(b)).

### 3.4. Ovarian Morphological Changes

**Figure 6:**
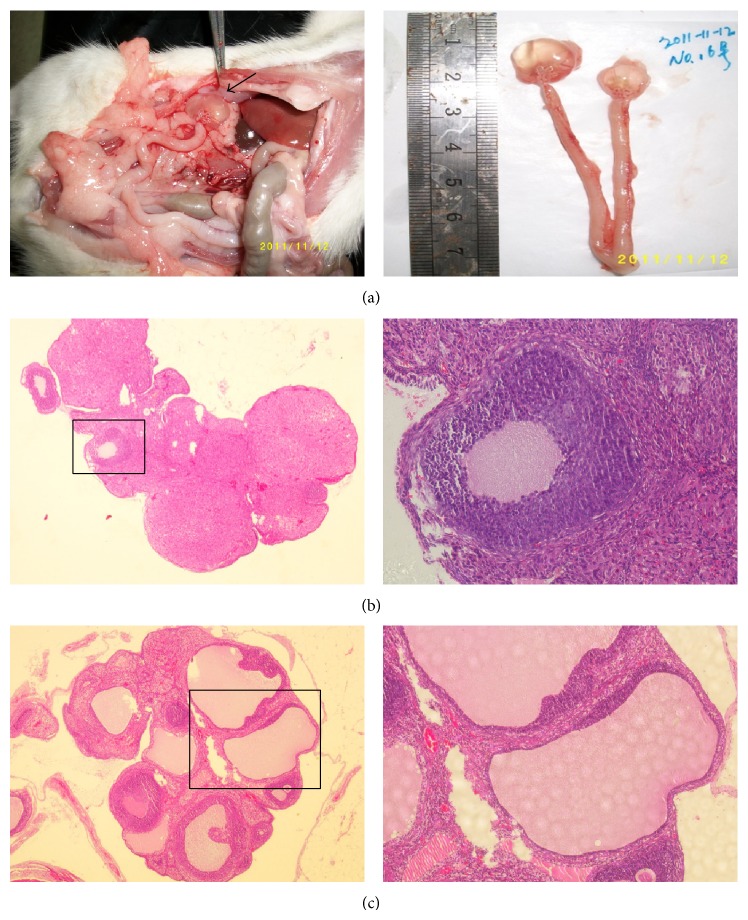
Macroscopic view and pathomorphological changes of rats' ovaries at the end of 16 weeks. (a) Left: macroscopic view of PCOS-induced ovaries (arrow) in rats. Right: the gross morphology of typical ovary and uterus. (b) Representative pathological section for L/D group; magnification is 40x (left) and 200x (right). (c) Representative pathological section for L/L group; magnification is 40x (left) and 100x (right).

We observed enlarged ovaries in the L/L group, and transparent fluid was visible through the surface layer tissue (Figure 6(a)).

Histopathological examination of ovarian tissue samples showed polycystic ovarian tissue formation in the L/L group, while the control group showed normal ovarian tissue histology. In the L/L group ovaries, thickening of the surface albuginea, under which there were many follicles in different phases, including atretic follicles and cystic dilating follicles, as well as fewer layers of granular cells and missing oocytes and corona radiating within the follicles, was present (Figure 6(c)). Ovaries in the control group showed multiple luteal, preantral, and antral follicles. The granular cells within the follicles showed multiple layers (Figure 6(b)). The incidences of this phenomenon were 0 of 12 in the L/D group and 10 of 12 (83.3%) in the L/L group (Table 3, L/D versus L/L, *P* = 6.73*E* − 5).

### 3.5. Elevated Testosterone Levels in Serum

**Figure 7:**
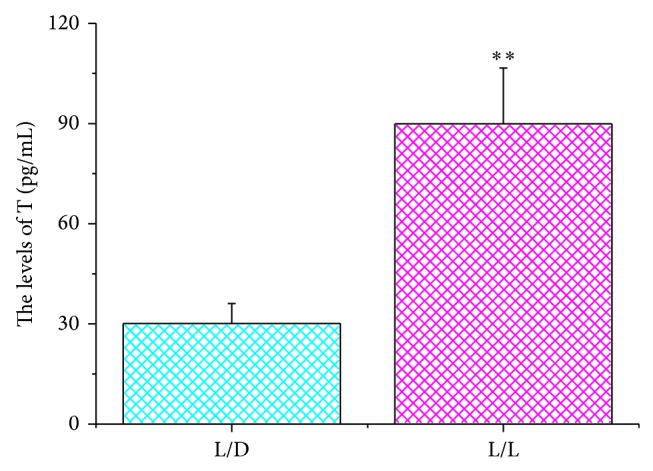
The levels of testosterone in rats' serum in different groups. The concentration of testosterone among rats in the continuous light group was greater than that in the control group (^∗∗^ *P* < 0.01, L/D group versus L/L group).

We compared the serum testosterone levels of the control and experimental groups and found that serum testosterone levels were significantly increased in the experimental group (30.11 ± 5.98 pg/mL versus 89.91 ± 16.72 pg/mL, *P* = 0.00353, *n* = 15) (Figure 7).

## 4. Discussion

In the present study, we first observed that serum testosterone levels of female rats increased in a continuous light environment; and also, our results show that body weights decreased in female rats in a continuous light environment with both ovarian and uterine augmentation; the estrous cycle in L/L rats was disordered, and polycystic ovary-like changes occurred, accompanied with fur loss and lethargy.

Steroid hormones play an important role in the effects of daylight changes. The hypothalamic suprachiasmatic nucleus (SCN) is the locus of a master clock that expresses androgen receptor (AR) in humans and regulates circadian rhythms in physiology and behavior. Research found that there is a correlation between plasma androgen levels and sleep onset in males, clock gene proteins expression in the SCN in response to the onset of environmental light [15, 16]. Testosterone concentration was positively correlated with SCN AR expression. Androgen can modulate SCN responsiveness to light and can modulate SCN timekeeping in a dose-dependent manner [17]. In mice, SCN restricts AR-containing cells and receives photic cues from the retina, and AR occurs in approximately half of the SCN neurons that respond directly to an acute light pulse by expressing FOS [18]. The SCN regulates the phasic release of hormones as well as the timing of the preovulatory LH surge necessary for ovulation in females [15]. In golden hamsters, the behavioral effects of androgen from seasonal changes in day length act are regulated via a pineal-dependent mechanism [19]. Meanwhile, males in the continuous dark condition (D/D) had higher serum androgen levels than in the L/L and L/D, while males in the L/L had higher serum prolactin levels [20]. Mice with a higher circulating testosterone concentration had more precise clocks and ran much faster, but for a shorter duration, than their counterparts with a low testosterone concentration. The LH surge is controlled by the anteroventral periventricular nucleus (AVPV) Kiss1 neurons, whose activity is gated by SCN signals in an estradiol- (E2-) dependent manner [21], and rats under L/L conditions induced a decrease in the serum estradiol level of rats in proestrus and an increase in rats in estrus [22]. Our study showed that serum testosterone levels of female rats increased obviously in a continuous light environment firstly, and it proves directly that the disorder of light conditions leads to the hormone level changes in female rats. This is a direct evidence which suggests the possibility that erratic lifestyle factor and bad sleep habits can lead to the onset of PCOS.

Earlier investigations have reported that the endometrium is thinner in women with PCOS and oligoamenorrhea [23], and, thus, the uteri are relatively small in humans with PCOS. Six of 8 hamsters exposed to L/L had ova in their oviducts at autopsy, and they also had significantly larger uteri (*P* < 0.01) than hamsters exposed to L/D [24, 25]. We found enlarged uteri filled with pus; these changes may be related to recurrent uterine infection. In rats, a lack of ovulation may cause a decrease in the ability of the reproductive system to self-clean due to long-term, repeated bacterial invasion because of the neuroendocrine system disorders and the consequent decline in immunity.

Rats exposed to constant light experience inhibition of the pineal gland function [26]; pinealocyte cell activity increases in rats exposed to constant darkness and decreases in rats exposed to constant light [27]. For rats exposed to constant dark or dim light, melatonin levels are completely suppressed [28]. In mammals, melatonin is synthesized by the pineal gland, and its synthesis is under direct control of the central circadian pacemaker that is located in the SCN of the hypothalamus [29]. Melatonin plays a role in the maintenance of proper follicular function and is thus important for ovulation and progesterone production [30]. Experiments in animals have confirmed beneficial effects of melatonin administration on oocyte maturation and embryo development [31]. Melatonin can reduce oxidative stress and contribute to oocyte maturation, embryo development, and the luteinization of granulosa cells. Melatonin can increase fertilization and pregnancy rates [32]. L/L induces constant estrous anovulatory (CEA) syndrome and blockage of pineal gland activity. Chronic treatment with melatonin is able to overcome the anovulatory state in approximately 70% of L/L-CEA rats, and the luteinizing effect of melatonin is significantly counteracted either by feeding the animals a tryptophan-poor diet or by injecting methiothepin (a blocker of central serotoninergic receptors). Melatonin elicits luteinization in L/L-CEA rats through the brain serotoninergic system [33]. Exposure to L/L regimens accelerates age-related switch-off of the estrous function in females [34].

Rats become stressed and anxious as a result of major changes in their environment. Constant light may lead to partial sleep deprivation; sleep deprivation is associated with physiological stress responses and an elevated cortisol response in the hypothalamic-pituitary-adrenal (HPA) axis [35]. Hair loss in this model indicates a neuro-endocrino-immune issue. In humans, autoimmune diseases are one of the most common reasons for alopecia areata, which can be considered a T-cell-mediated autoimmune disease [36]. In a rat model of alopecia areata, organ-specific autoimmune disease has been shown to have an important role in hair growth [37]. However, many hormones, and especially testosterone, influence hair growth. Androgenetic alopecia is the most common form of hair loss in men and women [38]. A testosterone-induced alopecia animal model has been used to research hair loss and treatments [39]. In hair follicles, the androgen binds to androgen receptors and exerts its effect directly [40]. Abnormal levels of androgen in our experiment may cause fur loss in rats. The experimental rats may also have disorders of the immune system that lead to fur loss.

## 5. Conclusions

In summary, the continuous light exposure-induced PCOS rat model simulates human sleep disorder. In this environment, rats appeared to experience pathologically and physiologically high androgen levels. This model simulates PCOS in human beings and is more consistent with the natural disease state. The pathologic physiological phenomenon demonstrated in this model may elucidate new mechanisms of PCOS. Poor sleep habits or negligence of sleep hygiene may be an important lifestyle factor in pathogenesis of PCOS.

## Figures and Tables

**Table 1:** Incidence of estrous cycle disorders at the end of 4 weeks of continuous light.

Groups	No. of rats with ECDs	No. of rats without ECDs	Total	Incidence	*P* value
L/D	0	12	12	0%	1.346*E* − 3
L/L	8	4	12	66.7%	

Total	8	16	24	—	

Abbreviation: ECDs: estrous cycle disorders.

**Table 2:** Incidence of estrous cycle disorders at the end of 16 weeks of continuous light.

Groups	No. of rats with ECDs	No. of rats without ECDs	Total	Incidence	*P* value
L/D	1	11	12	8.3%	9.6148*E* − 6
L/L	12	0	12	100%	

Total	13	11	24	—	

Abbreviation: ECDs: estrous cycle disorders.

**Table 3:** Incidence of polycystic ovary-like changes in histopathology at the end of 16 weeks of continuous light.

Groups	No. of rats with POLCs	No. of rats without POLCs	Total	Incidence	*P* value
L/D	0	12	12	0%	6.7304*E* − 5
L/L	10	2	12	83.3%	

Total	10	14	24	—	

Abbreviation: POLCs: polycystic ovary-like changes.
